# Toxic Potential and Metabolic Profiling of Two Australian Biotypes of the Invasive Plant Parthenium Weed (*Parthenium hysterophorus* L.)

**DOI:** 10.3390/toxins12070447

**Published:** 2020-07-10

**Authors:** Ali Ahsan Bajwa, Paul A. Weston, Saliya Gurusinghe, Sajid Latif, Steve W. Adkins, Leslie A. Weston

**Affiliations:** 1 School of Agriculture and Food Sciences, The University of Queensland, Gatton, QLD 4343, Australia; ali.bajwa@dpi.nsw.gov.au (A.A.B.); s.adkins@uq.edu.au (S.W.A.); 2 The Centre for Crop Science, Queensland Alliance for Agriculture and Food Innovation, The University of Queensland, Gatton, QLD 4343, Australia; 3 Weeds Research Unit, NSW Department of Primary Industries, Wagga Wagga, NSW 2650, Australia; 4 Graham Centre for Agricultural Innovation (Charles Sturt University and NSW Department of Primary Industries), School of Agricultural and Wine Sciences, Charles Sturt University, Wagga Wagga, NSW 2678, Australia; pweston@csu.edu.au (P.A.W.); sgurusinghe@csu.edu.au (S.G.); slatif@csu.edu.au (S.L.); 5 School of Animal and Veterinary Sciences, Charles Sturt University, Wagga Wagga, NSW 2650, Australia

**Keywords:** *Parthenium hysterophorus*, allelopathy, biological invasions, cytotoxicity, parthenin, metabolomics

## Abstract

Parthenium weed (*Parthenium hysterophorus* L.) is an invasive plant species in around 50 countries and a ‘Weed of National Significance’ in Australia. This study investigated the relative toxicity of the leaf, shoot and root extracts of two geographically separate and morphologically distinct biotypes of parthenium weed in Queensland, Australia. Parthenium weed exhibited higher phytotoxic, cytotoxic and photocytotoxic activity in leaf tissue extracts in contrast to shoot and root. The germination and seedling growth of a dicot species (garden cress) were inhibited more than those of a monocot species (annual ryegrass) using a phytotoxicity bioassay. The cytotoxicity of leaf extracts was assessed in a mouse fibroblast cell suspension assay and increased under high ultraviolet A(UV-A) radiation. A major secondary metabolite, parthenin, was found in abundance in leaf extracts and was positively correlated with cytotoxicity but not with photocytotoxicity or phytotoxicity. Ambrosin and chlorogenic acid were also detected and were positively correlated with germination inhibition and the inhibition of radicle elongation, respectively. In addition, other currently unidentified compounds in the leaf extracts were positively correlated with phytotoxicity, cytotoxicity and photocytotoxicity with two to three molecules strongly correlated in each case. Both parthenium weed biotypes investigated did not differ with respect to their relative toxicity, despite their reported differences in invasive potential in the field. This suggests that secondary chemistry plays a limited role in their invasion success.

## 1. Introduction

Invasive alien plant species are a global threat to biodiversity, ecosystem function and agricultural productivity [1]. Parthenium weed (*Parthenium hysterophorus* L.) is a highly invasive plant that has invaded 50 countries in Africa, Asia, and Oceania, where it infests forests, grazing lands, crop production systems and urban landscapes [1,2]. It is listed among the world’s most important invasive weeds due to its impacts on the natural and agricultural environment, including the loss of yield and quality in agronomic crops, loss of pasture and livestock productivity, land devaluation, and as the causal agent of serious health problems in mammalian systems [1,3]. In Australia, parthenium weed is a ‘Weed of National Significance’ due to its negative impact on grazing lands and the livestock industry [2]. It has been estimated to generate losses of AUD 16.8 million to the Queensland beef industry in the 1970s [4], with annual productivity losses predicted to be over $110 million by 2050 [2]. These losses were associated with the suppression of pasture growth and quality due to parthenium weed infestation, along with both direct and indirect effects on animal health [2,4]. Similar negative impacts have been reported from numerous countries in its invasive range [2].

Parthenium weed is also noted to be allelopathic and is associated with successful plant invasion through the production of novel chemical weapons [3]. There are numerous reports on the phytotoxic or allelopathic potential of the species, but many provide an exaggerated account of this important ecological attribute. Nevertheless, a significant number of studies have evaluated the phytotoxic potential of parthenium weed with precision. Parthenium weed has been reported to suppress the germination and early growth of many crops, pasture and weed species [5,6,7,8,9,10,11]. Parthenium weed has also been reported to release allelochemicals directly as root exudates in the rhizosphere, or by the leaching of fresh or decomposing residues, the washing of leaves by rain, or as volatiles released over time from aerial plant portions [5,7,8].

The phytochemistry of parthenium weed has also been investigated, with a diverse set of metabolites identified in its leaves, shoots, roots and inflorescences [1,2]. Several secondary metabolites have been associated with stress tolerance [12] or allelopathic activity [10,13]. One of the most abundant and well characterised bioactive metabolites is the sesquiterpene lactone parthenin. Several studies have attributed the phytotoxicity of parthenium to the presence of parthenin [8,10,11]; however, other reports have suggested that parthenin is only partially responsible for its phytotoxicity [13,14]. Several sesquiterpene lactones and phenolics isolated from parthenium weed have been associated with phytotoxicity [8,15,16,17,18]. Flavonoids and alkaloids have also been reported as allelochemicals produced by parthenium weed [19], but most have been suggested to be minor contributors to its overall phytotoxicity.

In addition to its deleterious impacts on crop health, parthenium weed can also be detrimental to animal and human health [20]. Although animals do not preferentially ingest the plant due to its low palatability, plant parts consumed by livestock when mixed with fodder or during grazing are toxic [2]. Parthenium weed has been associated with contact dermatitis, photosensitisation, asthma, loss of appetite, and indigestion in several animal species, including cattle (*Bos taurus* L.), buffalo (*Bubalus bubalis* L.), sheep (*Ovis aries* L.), goat (*Capra hircus* L.), camel (*Camelus bactrianus* L.) and horse (*Equus caballus* L.) [4,20,21,22]. The meat of animals fed on parthenium weed was also found to be tainted, with significant impacts on flavour and quality [21]. In severe cases, cattle and buffalo calves succumbed to parthenium weed toxicity [22].

In Australia, parthenium weed occurs as two notable biotypes which differ with respect to their geographic location, introduction history, invasive ability and spread [2]. The first introduction occurred in the mid-1940s in Toogoolawah (southeast Queensland) and has not spread far from its point of introduction; it is now referred to as the Toogoolawah biotype. The second introduction occurred in 1958 in Clermont (central Queensland); this population has since spread widely across central and southern Queensland and is known as the Clermont biotype [2].

Previous studies have shown that the Clermont biotype has higher germinability in collected seed lots, a more vigorous growth habit, more efficient resource acquisition capacity and superior adaptive ability when compared to the Toogoolawah biotype [23,24]. However, recent studies have suggested that no significant genetic diversity is present in the two contrasting biotypes [25]. Shi and Adkins [6] compared both biotypes for the phytotoxic potential of their leaf litter and found no significant differences in the allelopathic activity. Parthenium weed was ranked as a moderately allelopathic species when compared to some well-known highly allelopathic plant species [6]. However, it is important to note that this study evaluated the allelopathic effects of dried leaf litter of parthenium weed, not the living plant.

Clearly, significant efforts have been made in the past to study the secondary chemistry of parthenium weed in relation to its toxic potential. However, the lack of appropriate analytics has hindered progress in correlating its presence with observed toxicity due to infestation. In recent years, advanced metabolomics techniques to assess the chemical composition of plants in relation to their bioactive constituents have gained popularity as strong and reliable tools [26] for the characterisation of key metabolites. For example, liquid chromatography (LC) or ultra-high pressure liquid chromatography (UHPLC) coupled with mass spectrometry (MS) have been successfully used for the targeted and non-targeted profiling of bioactive metabolites of invasive plant species such as *Echium* spp. [27,28]. Moreover, statistical procedures such as stepwise linear regression between the metabolic features and bioactivity of plant extracts have been found to be powerful tools for facilitating the identification of causal metabolites [26,28]. Therefore, it is critical to take a fresh look at parthenium biology with respect to its allelopathic potential and cytotoxicity in animal tissues using these techniques.

The two Australian biotypes of parthenium weed provide a logical framework for comparative analysis and thus, the present study was carried out (1) to determine the toxic effects of plant extracts of both biotypes against animal tissues and seed germination and elongation, (2) to determine the role of the major secondary metabolites of parthenium weed on the bioactivity (phytotoxicity, photocytotoxicity and cytotoxicity) of its crude extracts, and (3) to identify other potentially bioactive compounds of interest through non-targeted metabolomics. It was hypothesised that (1) the invasive biotype of parthenium weed, Clermont, may be more phytotoxic compared to the non-invasive biotype, Toogoolawah, and this might be a contributing factor for the invasion success of the former, and (2) sesquiterpene lactones such as parthenin might be the major secondary metabolites responsible for parthenium weed toxicity to animals and plants. The results of this study will thus significantly improve our understanding of the mechanism(s) associated with parthenium weed invasion, its secondary chemistry, potential for interference with crop and pasture production and its impact on mammalian health.

## 2. Results

### 2.1. Phytotoxicity

The crude shoot and root extracts of parthenium weed caused no significant inhibition of garden cress seed germination, radicle elongation and hypocotyl elongation at moderate dosages in comparison to the methanol control (Table S1). However, the leaf extracts of parthenium weed caused a highly significant (*p* < 0.001) inhibition of seed germination and radicle and hypocotyl elongation of both garden cress and annual ryegrass (Table S2). The leaf extracts of Clermont and Toogoolawah biotypes differed significantly (*p* < 0.05) with respect to the inhibition of germination and hypocotyl elongation in garden cress, but not for radicle elongation (Table S2). Clermont leaf extracts were 19 and 9% more inhibitory to garden cress germination and hypocotyl elongation, respectively, as compared to Toogoolawah leaf extracts. However, the leaf extracts of the two biotypes did not differ in their toxicity to annual ryegrass (Table S2).

**Figure 1:**
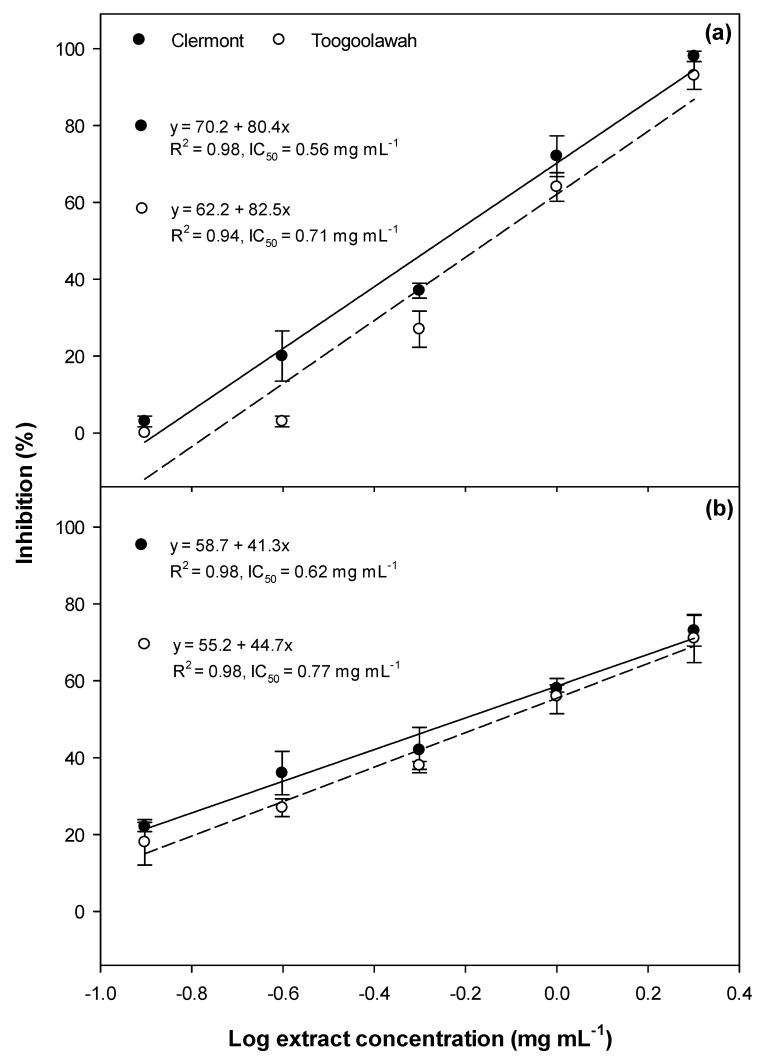
Germination inhibition of (**a**) the garden cress and (**b**) the annual ryegrass seeds by the leaf extracts of the Clermont and Toogoolawah biotypes of parthenium weed. The log of the actual concentrations ranging from 0.12 to 2.00 mg mL^−1^ was taken to fit the linear regression curves. Error bars represent ± one standard error.

**Figure 2:**
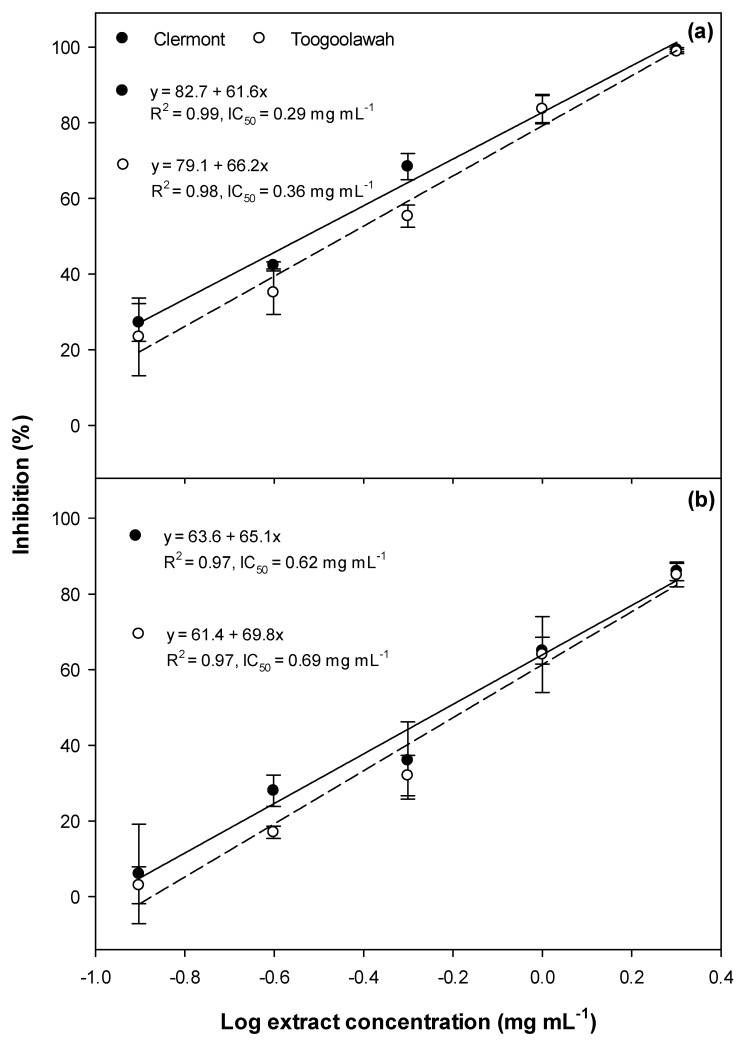
Radicle elongation inhibition of (**a**) the garden cress and (**b**) the annual ryegrass seedlings by the leaf extract of the Clermont and Toogoolawah biotypes of parthenium weed. The log of the actual concentrations ranging from 0.12 to 2.00 mg mL^−1^ was taken to fit the linear regression curves. Error bars represent ± one standard error.

**Figure 3:**
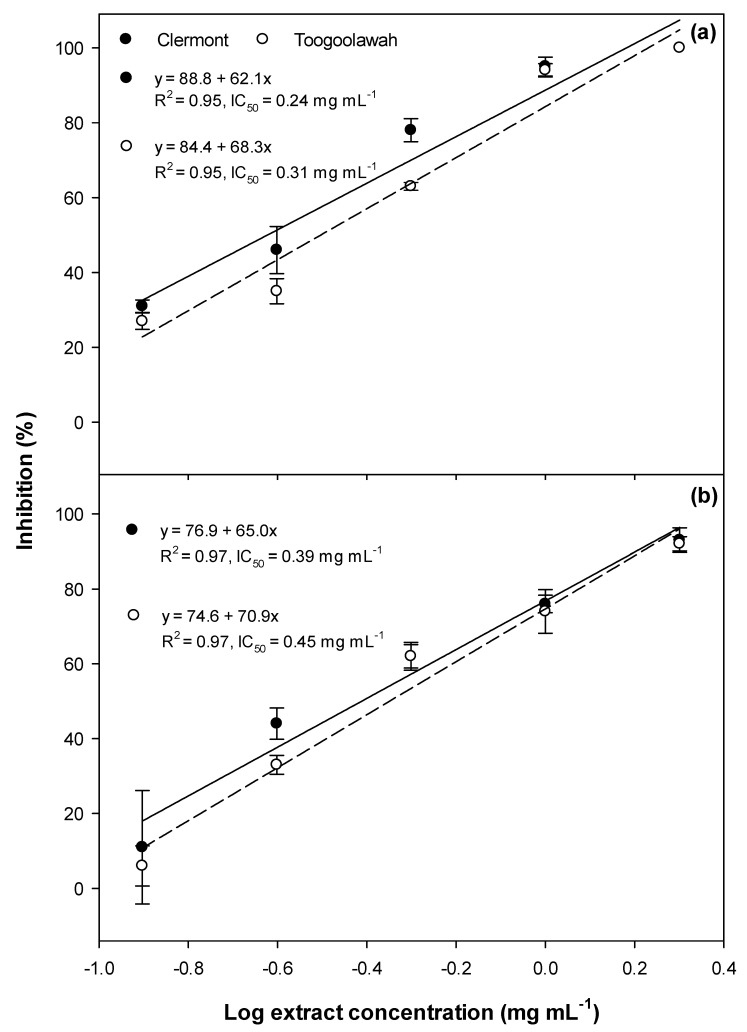
Hypocotyl elongation inhibition of (**a**) the garden cress and (**b**) the annual ryegrass seedlings by the leaf extract of the Clermont and Toogoolawah biotypes of parthenium weed. The log of the actual concentrations ranging from 0.12 to 2.00 mg mL^−1^ was taken to fit the linear regression curves. Error bars represent ± one standard error.

Significant inhibition of germination (Figure 1), as well as radicle (Figure 2) and hypocotyl elongation (Figure 3), were noted in both garden cress and annual ryegrass treated with leaf extracts of both biotypes. However, the dose–response curves revealed that the Clermont leaf extracts had lower IC_50_ (concentration required to inhibit 50 response compared to positive control ) values for germination as well as the radicle and hypocotyl elongation of garden cress and annual ryegrass, as compared to those of Toogoolawah (Figure 1, Figure 2 and Figure 3). The inhibitory effects increased linearly with the increasing concentration of extracts (Figure 1, Figure 2 and Figure 3). Overall, the highest concentration of leaf extract studied (2 mg mL^−1^) provided the greatest inhibition of garden cress germination (96%), radicle elongation (99%) and hypocotyl elongation (100%) when compared to the methanol control. Similarly, the leaf extract at this concentration also resulted in the significant inhibition of annual ryegrass germination (72%), radicle elongation (86%) and hypocotyl elongation (93%) as compared to the methanol control. The interactions between the biotype and the extract concentration for all these parameters were non-significant for all the plant tissue types (Tables S1 and S2).

**Figure 4:**
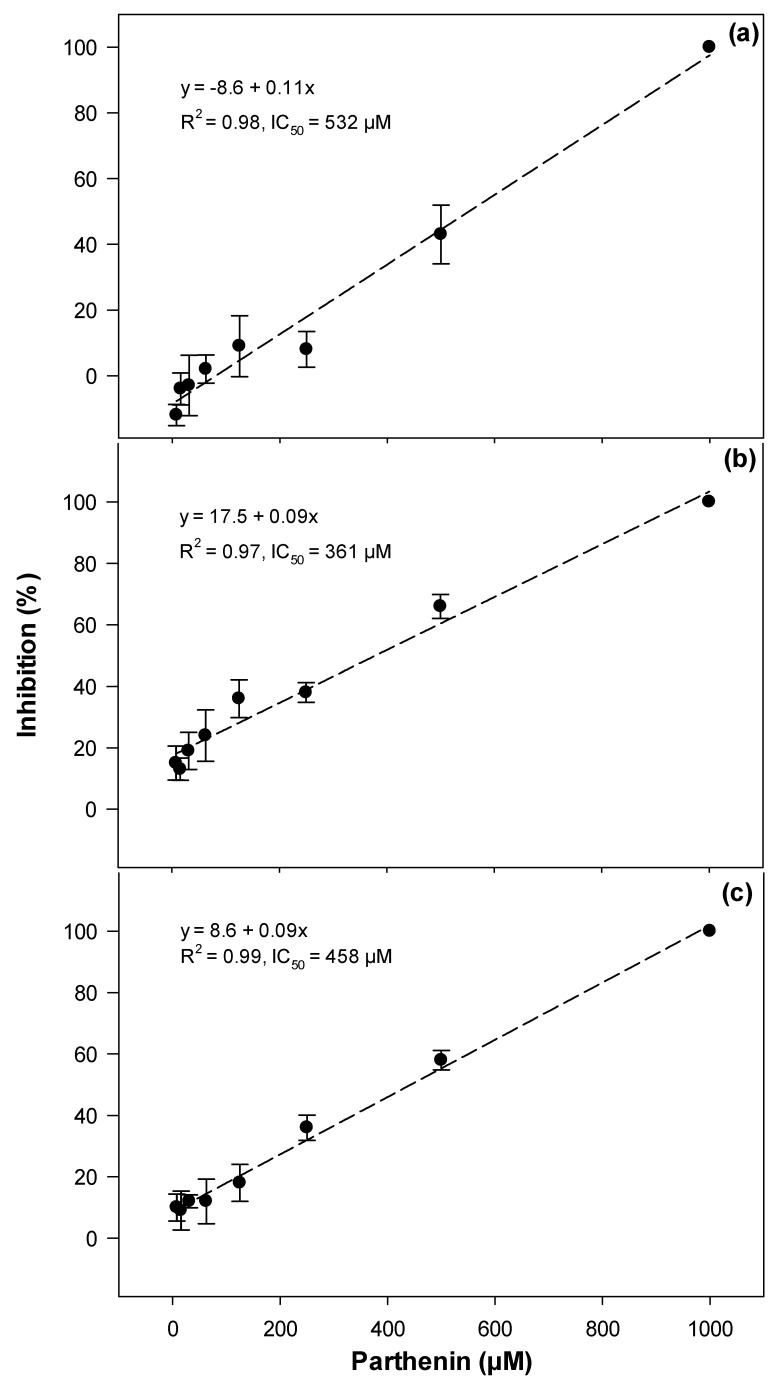
The inhibition of (**a**) seed germination, (**b**) radicle elongation and (**c**) hypocotyl elongation of the garden cress by parthenin. Error bars represent ± one standard error.

Parthenin treatment also resulted in the significant inhibition of garden cress seed germination as well as radicle and hypocotyl elongation when compared to the methanol control (Figure 4). Garden cress seed germination was somewhat affected by parthenin, with an IC_50_ value of 532 μM, whereas hypocotyl and radicle elongation were most impacted with the IC_50_ values of 458 and 361 μM of parthenin, respectively (Figure 4).

### 2.2. Cytotoxicity and Photocytotoxicity

**Figure 5:**
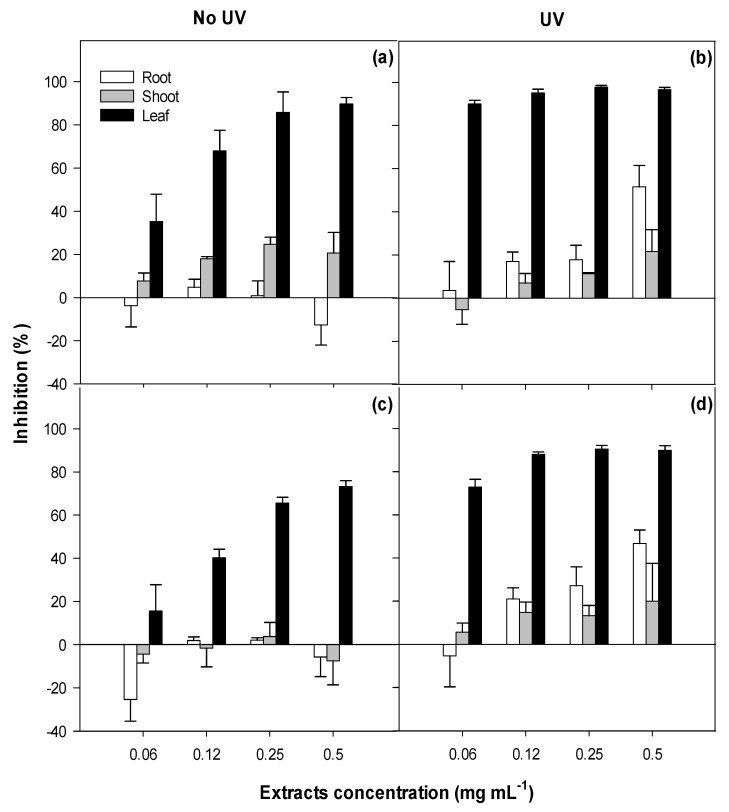
Cytotoxicity (no UV; under dark conditions) and photocytotoxicity (UV; under high ultraviolet A radiation) against the murine fibroblasts following the treatment with the leaf, shoot and the root extracts of (**a**,**b**) the Clermont and (**c**,**d**) the Toogoolawah biotypes of parthenium weed. Error bars represent ± one standard error.

**Figure 6:**
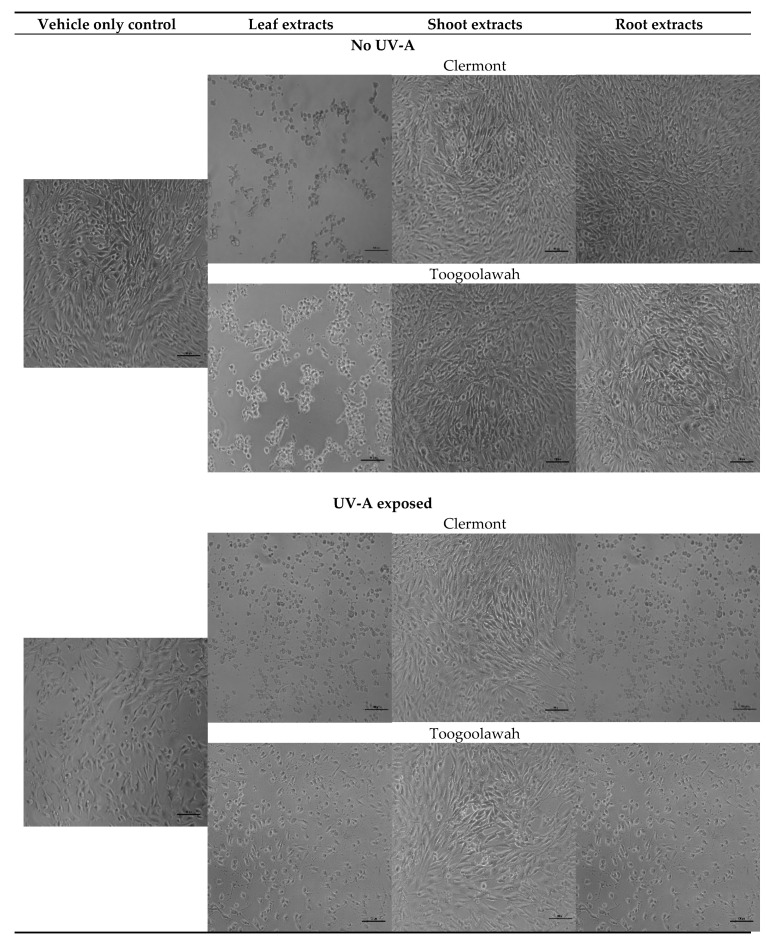
Typical morphology of the mouse fibroblasts 48 h after treatment with the leaf, shoot or the root extracts of the Clermont and Toogoolawah biotypes of parthenium weed at the concentrations of 0.5 mg mL^−1^ in the absence or presence of UV-A radiation. Non-viable cells are exhibiting typical apoptotic features including nuclear condensation and cell shrinkage. Scale bar = 100 µm.

The viability of the NIH3T3 fibroblasts was negatively and rapidly impacted by the increasing concentrations of leaf extracts in a dose-dependent manner (Table S3; Figure 5). In contrast, the shoot and root extracts had no significant impact on cell viability, with root extracts at the lowest concentration (0.0625 mg mL^−1^) promoting cell viability (14%) when compared to the untreated control (Figure 5). The cytotoxicity of leaf extracts differed between the two biotypes (*p* < 0.001). The viability of murine fibroblasts following the exposure under high ultraviolet A (UV-A) radiation differed (*p* < 0.001) between the tissue types, but not the biotypes (Table S3). The leaf extracts caused the highest reduction in cell viability (90%), followed by root extracts (22%) and shoot extracts (11%) (Figure 5). In summary, only the leaf extracts of both biotypes of parthenium weed presented strong cytotoxicity (up to 81%) and photocytotoxicity (up to 90%) within 48 h after treatment (Figure 5 and Figure 6).

**Figure 7:**
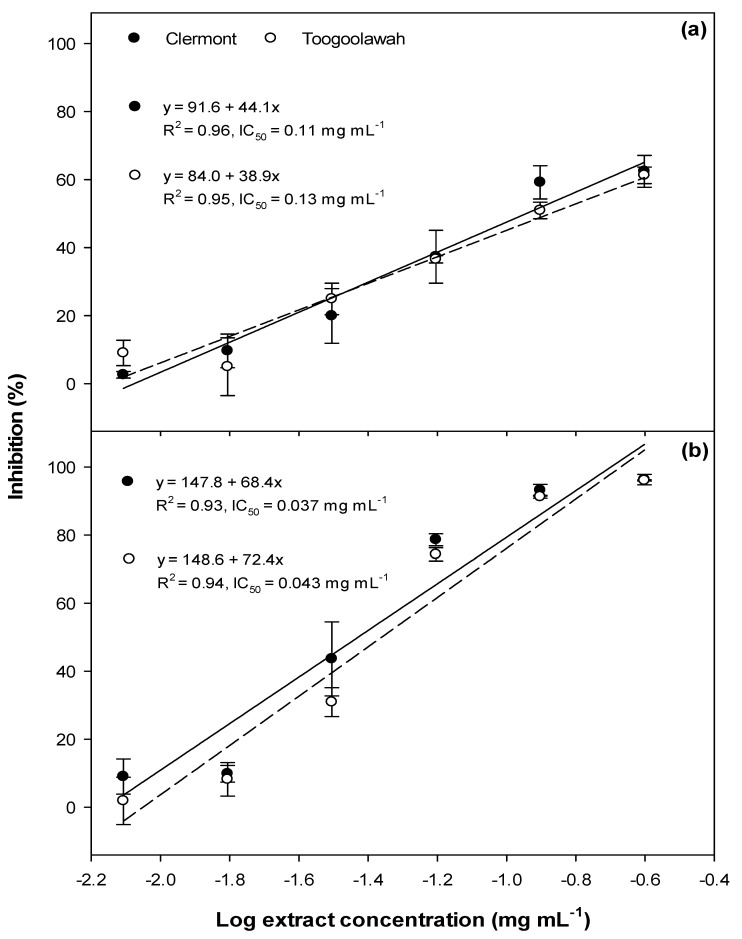
The (**a**) cytotoxicity and (**b**) photocytotoxicity of the leaf extracts of the Clermont and Toogoolawah biotypes of parthenium weed against the murine fibroblasts. The log of the actual concentrations ranging from 0.008 to 0.25 mg mL^−1^ was taken to fit the linear regression curves. Error bars represent ± one standard error.

The photocytotoxic assessment of the leaf extracts ranging from 0.06 to 0.5 mg mL^−1^ was insufficient to deduce a linear relationship between the extract concentration and cell viability. Hence the experiment was repeated with a broader range of extract concentrations ranging from 0.008 to 0.25 mg mL^−1^. A strong linear relationship was observed between the extract concentration and the reduction of cell viability, with or without exposure to UV-A radiation (Figure 7). The leaf extracts of both biotypes of parthenium weed did not differ significantly (*p* = 0.858; Table S4) in their cytotoxicity, with the IC_50_ values of 0.11 and 0.13 mg mL^−1^ for the Clermont and Toogoolawah biotypes, respectively (Figure 7a). The viability of the fibroblasts further decreased following the exposure to UV-A radiation; however, the two biotypes again did not differ significantly (*p* = 0.107; Table S4), with the IC_50_ values being reduced to 0.037 and 0.043 mg mL^−1^ for the Clermont and Toogoolawah biotypes, respectively (Figure 7b).

### 2.3. Quantitation of Major Known Secondary Metabolites

**Figure 8:**
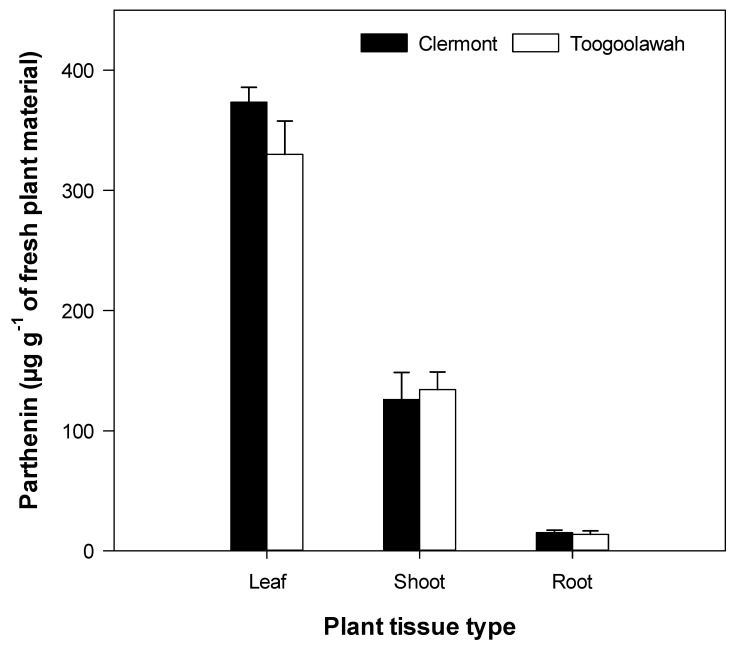
The quantity of parthenin in the leaf, shoot or the root tissues of the Clermont and Toogoolawah biotypes of parthenium weed. Error bars represent ± one standard error.

**Figure 9:**
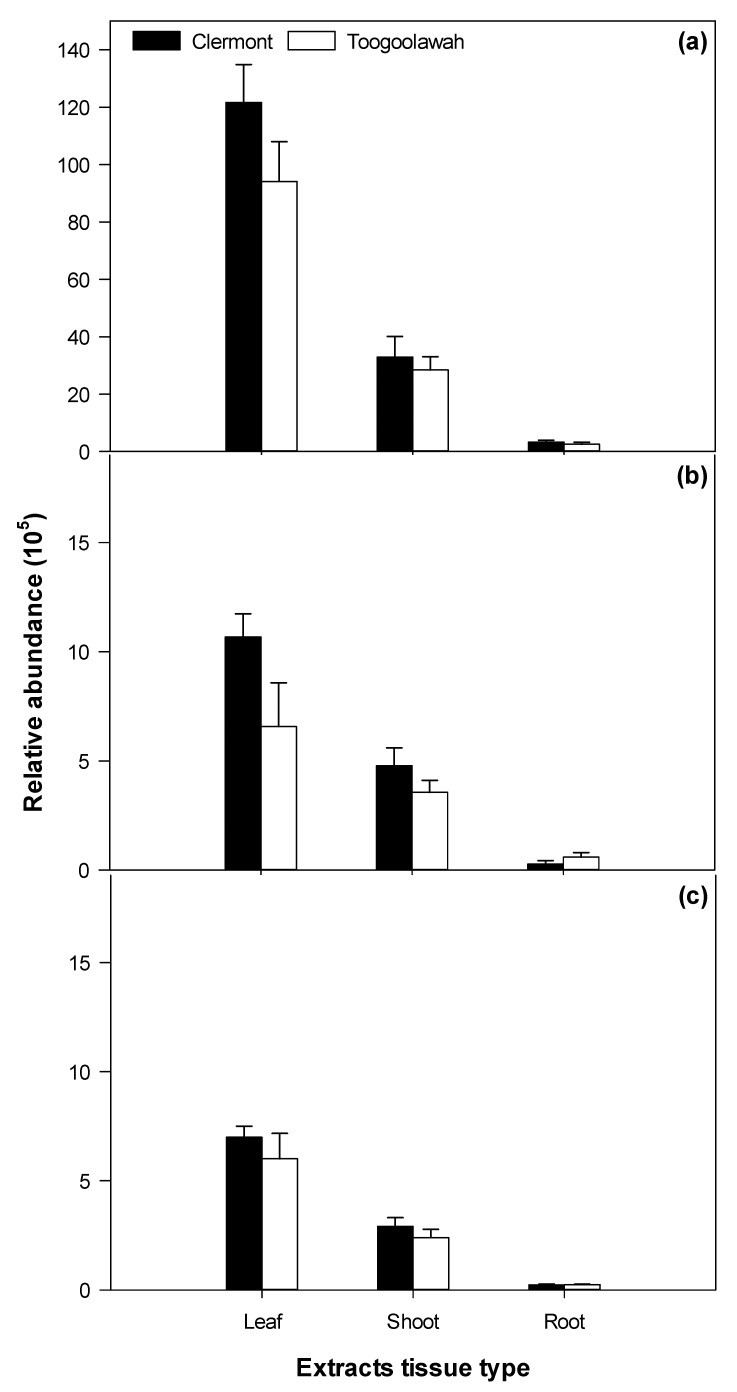
The relative abundance of (**a**) coronopilin, (**b**) ambrosin and (**c**) damsin in the leaf, shoot or the root extracts of the Clermont and Toogoolawah biotypes of parthenium weed. Error bars represent ± one standard error.

Only four of the previously reported sesquiterpene lactones of importance (parthenin, coronopolin, ambrosin, damsin) and one phenolic acid (chlorogenic acid) were positively detected in abundance in the leaf, shoot and the root tissue extracts of parthenium weed. Parthenin was the most abundant of all metabolites detected and varied significantly (*p* < 0.001) by tissue type (leaf, shoot and root) but not between the biotypes (Table S5). The relative abundance of coronopolin, ambrosin and damsin also differed significantly (*p* < 0.001) by tissue; however, chlorogenic acid was equally abundant (*p* = 0.575) across all tissues (Table S5). Parthenin was by far the most abundant of the sesquiterpene lactones, followed by coronopilin, ambrosin and damsin, respectively (Figure 8 and Figure 9). Parthenin was present in the highest quantity in the leaf tissues (352 µg g^−1^ of fresh weight) followed by the shoot and root tissues (Figure 8). The relative abundance of the other sesquiterpene lactones followed a similar pattern (Figure 9). However, the quantity of parthenin and the relative abundance of other metabolites clearly did not differ between the Clermont and Toogoolawah biotypes (Table S5).

### 2.4. Distribution of Unidentified Molecular Entities

**Figure 10:**
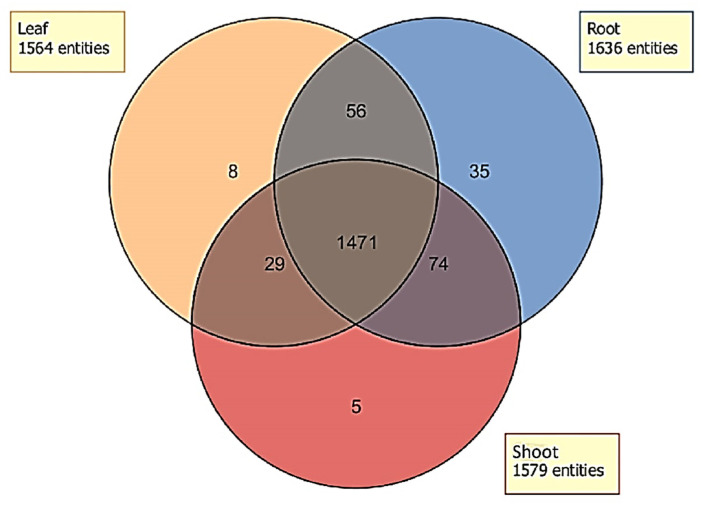
Venn diagram representing the distribution of the molecular entities detected in the leaf (orange), shoot (red) or the root (blue) extracts of parthenium weed.

Many molecular entities (including individual compounds and their fragments or derivatives) were detected in the extracts of various plant tissues of parthenium weed (Figure 10). No single entity was exclusive to either biotype, therefore the data for both biotypes were pooled to evaluate only the tissue types. The total number of entities differed in each tissue type (Figure 10). Although most entities were present in all three tissue types (1471), some entities were exclusively present in only one or two. For instance, eight, five and 35 compounds were exclusively present in the leaf, shoot and root extracts, respectively (Figure 10). Similarly, 29 entities were present in both the leaf and shoot extracts but absent in the root extracts, whereas the number of mutually shared entities between the shoot and root (74) or the leaf and root extracts (56) was much higher. However, the identification of these entities was generally not possible due to the lack of a match with known libraries and analytical standards for comparative purposes.

**Figure 11:**
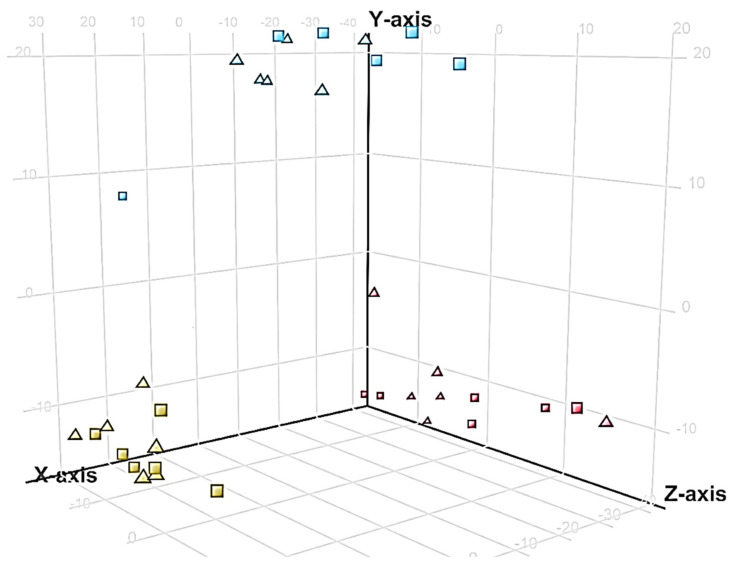
Principal component analysis (PCA) representing the spread of the different samples of the leaf (red), shoot (blue) and the root extracts (yellow) of the Clermont (triangles) and Toogoolawah (squares) biotypes of parthenium weed, based on the relative abundance of all the molecular features detected by LC–MS QTOF.

Molecular features differed among the leaf, shoot and the root extracts; however, both biotypes clustered together in the PCA plots, suggesting limited chemical differences between the biotypes (Figure 11).

### 2.5. Associations between Compound Abundance and Toxicity

**Figure 12:**
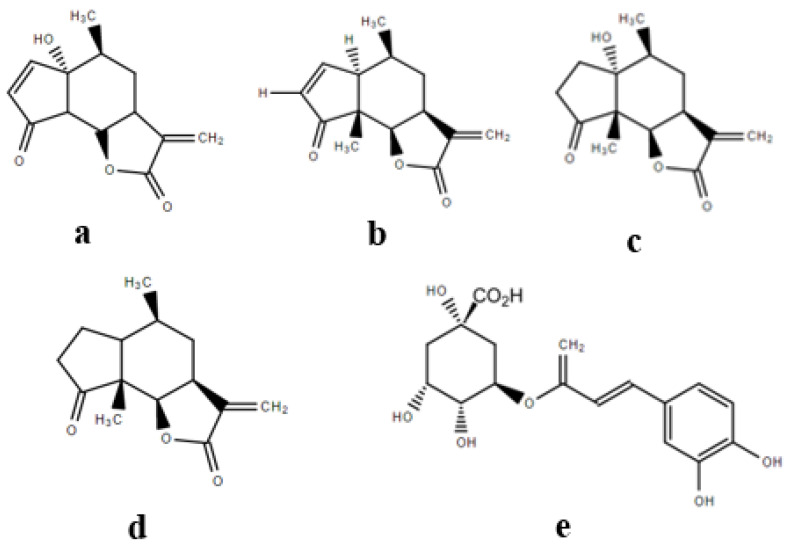
Chemical structure of the phytotoxic compounds (**a**) parthenin, (**b**) ambrosin, (**c**) coronopilin, (**d**) damsin and (**e**) chlorogenic acid in the associated parthenium weed extracts.

Simple correlations between the abundance of known metabolites (Figure 12) and phytotoxicity (inhibition of germination, radicle elongation and hypocotyl elongation of garden cress) ranged from 0.20 to 0.70, but only two identified metabolites were significantly and positively correlated with phytotoxicity (Table 1). Stepwise linear regression found ambrosin to be positively correlated with the inhibition of germination (R^2^ = 0.52, *p* = 0.012) and chlorogenic acid to be positively correlated with the inhibition of radicle elongation (R^2^ = 0.40, *p* = 0.041). Other sesquiterpene lactones previously reported in the literature (parthenin, coronopolin and damsin) were not found to be associated with phytotoxicity. In the assessment of animal cells, parthenin was significantly correlated with the observed cytotoxicity in murine fibroblasts in the dark (no UV-A) (R^2^ = 0.79, *p* < 0.001) but not in the presence of UV-A radiation, suggesting its potential lability under high light conditions.

A non-targeted analysis was conducted to take a more expansive look at the relative contributions of all the chemical constituents in the leaf extracts from each biotype of parthenium weed to the bioactivity assessed in the laboratory assays performed, using stepwise linear regression. The dependent variable was the bioactivity measure (inhibition of germination, inhibition of radicle elongation, inhibition of hypocotyl elongation or cytotoxicity) and the independent variables included the molecular entities extracted by molecular feature extraction from the chromatograms of individual samples via Profinder. A total of 1275 entities were detected and allowed to enter or leave the model. Although the regression analysis identified three metabolites that accounted for up to 90% of the variation in each bioassay (Table 2), the metabolites of importance varied for each of the bioassays. The identities of several of these metabolites could not initially be determined and did not match the retention time and accurate mass of standards, or the other metabolites previously described in the literature. Numerous metabolites appeared to be related derivatives of carboxylic acids, but further investigation will be required for positive identification.

A separate analysis was conducted for photocytotoxicity using the leaf extracts of each of the two biotypes. These samples were processed separately with Profinder before conducting stepwise linear regression; a total of 1547 molecular entities were allowed to enter or leave the model. A single component (Compound 1242) accounted for 84% of the variation in photocytotoxicity, and the addition of an additional component (Compound 1075) brought this figure to 99% (Table 2). These two compounds currently remain unidentified.

## 3. Discussion

The results of this study revealed that the leaf tissue extracts of parthenium weed are toxic to garden cress, annual ryegrass, and murine fibroblasts; however, the cytotoxicity was enhanced upon the exposure to UV-A radiation. which suggests the presence of potential photosensitizing agents in these extracts [29]. Shoot and root extracts showed limited to no activity in each assay when compared to the respective controls. This clearly suggests that the toxic secondary metabolites of interest are located mainly in the parthenium weed leaf tissues [10,30]. Parthenium weed biotypes did not differ significantly with respect to toxicity, except for phytotoxicity in garden cress, as the Clermont biotype was slightly more toxic than the Toogoolawah biotype. These findings suggest that plant toxicity is not likely to be closely associated with their contrasting invasion success [6].

### 3.1. Phytotoxicity/Allelopathy

The higher phytotoxic potential of the leaf extracts of parthenium weed noted in this study is consistent with other reports in the literature. Leaf extracts or litter/residues of aboveground parts of parthenium weed exhibited allelopathic effects on a range of monocot and dicot crop, pasture or weed species [5,6,10,11,31]. Aslam et al. [31] reported that the leaf extracts and leaf residues of parthenium weed were more inhibitory to the germination and early seedling growth of wild oat (*Avena fatua* L.) and little seed canarygrass (*Phalaris minor* Retz.), compared to the whole-plant or root extracts and/or residues in the Petri dish and soil bioassays. Parthenium weed allelopathy in field studies has been previously associated with above-ground plant parts [10,13]. However, the phytotoxicity observed in the controlled laboratory assays with extracts at high concentrations is frequently not indicative of allelopathic activity under field conditions [32], and our findings are not suggestive of such.

The higher sensitivity of the dicot (garden cress) compared to the monocot species (annual ryegrass) assayed in this study is potentially due to the differences in selectivity associated with the chemical composition of the extracts. The potential differences in the seed structure, seed coat texture and membrane permeability of the two species might also have played an important role in their differential sensitivity. In a previous study, the germination and early growth of billygoat weed (*Ageratum conyzoides* L.) were most sensitive to the crude leaf extracts of parthenium weed as well as to parthenin as compared to monocot species, barnyard grass (*Echinochloa crus-galli* (L.) P. Beauv.), African love grass (*Eragrostis curvula* (Schrad) Nees) and teff (*Eragrostis tef* (Zucc.) Trotter) [13]. However, selectivity differences exhibited by parthenium weed extracts suggest that additional study may be required [13].

The leaf tissues of parthenium weed have been reported to possess several secondary metabolites associated with its phytotoxic potential [30,33]. In the present study, the relative abundance of most metabolites of interest was higher in the leaf extracts than in the root or stem, which is consistent with the observed phytotoxic potential. Sesquiterpene lactones were noted to predominate in the bioactive extracts of parthenium weed and other closely related species in *Asteraceae* [34,35,36].

Numerous unrelated sesquiterpene lactones [35,36] have been reported as potent phytotoxins and may also play protective roles under biotic and abiotic stresses [35,36]. Several sesquiterpene lactones from sunflower (*Helianthus annuus* L.) including annuolide H, helivypolides F, and helieudesmanolide A were found to be extremely toxic to both monocot and dicot plant species at concentrations of 1 to 100 μM [36]. Sesquiterpene lactones are thought to be the derivatives of mevalonic acid, however, the exact pathway of their biosynthesis is not clear [35]. The α-methylene-γ-lactone moiety has been associated with the activity of most sesquiterpene lactones [36], which are known to inhibit the seed germination and seedling growth by cross reacting with the sulfhydryl-groups of amino acids, by inhibiting the cell division or by reducing the activities of important enzymes such as dehydrogenases, proteases, and peroxidases [37,38]. However, the sesquiterpenes under study in these experiments proved not to be particularly phytotoxic under the conditions evaluated, including parthenin as a purified standard.

Parthenin has remained the focus of numerous studies evaluating the allelopathic potential of parthenium weed [12,13,38]. It is produced and sequestered in the highest concentrations in the leaf trichomes of parthenium weed, but it is not transported to the roots [30,33]. Therefore, phytotoxicity exhibited by leaf extracts or leaf litter could be well associated with this chemical but potentially not with any root-related allelopathy of parthenium weed [13]. In fact, Belz, van der Laan, Reinhardt and Hurle [14] reported that parthenin degraded quickly in the soil due to microbial activity, and we also observed a lack of activity under high ultraviolet A (UV-A), suggesting its lability. Although parthenin was the most abundant compound in the leaf extracts, it was also not significantly correlated with the phytotoxicity observed in the present study. Parthenin also exhibited moderate phytotoxicity compared to potent allelochemicals such as sorgoleone and *m*-tyrosine which produced IC_50_ values as low as 10 and 8 μM, respectively [39,40]. Parthenin has exhibited auxin-like hormetic effects at low concentrations but the exact mechanism of bioactivity of this compound is unclear [13].

Another sesquiterpene lactone, ambrosin, was associated with the germination inhibition of the test species in this study; this compound is a well known allelochemical of parthenium weed [8]. Ambrosin has also been isolated from sea ragweed (*Ambrosia maritima* L.) and was found to be a strong inhibitor of germination, radicle elongation and the hypocotyl elongation of wild oat seeds [34]. We found ambrosin levels to be correlated with the inhibition of young seedling growth but not seed germination, similar to artemisinin and other sesquiterpene lactone analogues [34,37]. The enhanced activity of ambrosin could potentially be due to possibly greater absorption and transport through cellular membranes [41].

Chlorogenic acid, a phenolic compound, was correlated with the inhibition of radicle elongation in the present study; this compound has previously been identified as a potent allelochemical in many species including parthenium weed [8,42]. For example, chlorogenic acid was found to inhibit the growth of lettuce seedlings at concentrations above 100 μM [43]. We note that many phenolic compounds have been reported to play a major role in parthenium weed allelopathy, especially in the rhizosphere through direct root exudation or by leaching from the aerial parts [7,8]. Most phenolics of parthenium weed were reported to influence the germination and growth of other plants, not only through direct phytotoxicity but also through the manipulation of the physicochemical properties of soil and nutrient availability [7,8,44]. Although the higher solubility of phenolics in water also make their transport in plants easier [9], most do not exhibit strong phytotoxicity over more than a 24 h period as they are highly labile in soil conditions and therefore, the involvement of other potential secondary metabolites has been suggested [13].

Several unidentified metabolites in this study showed strong correlations with various measures of phytotoxicity, and our observations of their structure from mass fragmentation patterns and molecular weight suggest that these compounds could potentially be derivatives of parthenin, which was not unexpected because of the proclivity of sesquiterpenoids to undergo transformation in the environment [14]. Datta, et al. [45] reported that some derivatives of parthenin were more active than parthenin itself, and Belz, Reinhardt, Foxcroft and Hurle [13] found that the allelopathic activity of crude leaf extracts of parthenium was greater than that of parthenin, with the relative contribution of parthenin to the overall allelopathy of leaf extracts varying significantly for different test species. The balance of evidence suggests that parthenin is probably not the most active allelochemical in parthenium weed leaf extracts, as suggested by Belz, Reinhardt, Foxcroft and Hurle [13]. Various extracts or mixtures of sesquiterpene lactones and phenolic acids may act synergistically, contributing to the enhanced phytotoxicity of the crude leaf extracts of parthenium weed.

### 3.2. Cytotoxicity and Photocytotoxicity

Parthenium weed extracts exhibit stronger cytotoxicity under exposure to UV-A radiation, suggesting that this species causes photosensitization in animals and humans [29]. The lower cytotoxicity/photocytotoxicity exhibited by the shoot and root extracts as compared to the leaf extracts of parthenium weed may be associated with the significantly higher levels of parthenin in the leaf extracts. The literature suggests that the deposition of higher amounts of parthenin in leaf trichomes is a defence strategy of parthenium weed against herbivory [20]. The production of parthenin typically peaks at the start of flowering, a vital stage in the life of the plant when herbivory needs to be prevented [33]. This is the stage when the samples were collected in the present study and parthenin was also observed to be highly abundant in the leaf extracts.

The strong correlation between parthenin and cytotoxicity observed in this study is consistent with previous reports [15,46,47]. Parthenin itself was highly cytotoxic to bovine kidney cells by inhibiting the synthesis of RNA, DNA and key cellular enzymes within 24 h after exposure [47]. Similarly, other sesquiterpene lactones including ambrosin, coronopilin, tetraneurin A and hysterone D, and some acetylated pseudoguaianolides have been associated with the cytotoxicity of aerial parts of parthenium weed [15,48,49]. The α-methylene-γ-lactone moiety was suggested to be responsible for cytotoxicity for most of these compounds [49]. In addition to parthenin, two unidentified compounds were strongly correlated with cytotoxicity in the present study; these might be derivatives of parthenin or other unidentified sesquiterpene lactones. Interestingly, parthenin abundance was found to be uncorrelated with photocytotoxicity, but it is also possible that parthenin lability under light accounts for this lack of activity. The literature also suggests that cytotoxic and photocytotoxic activities may not be interrelated and that entirely different metabolites could be associated with each form of toxicity [29]. However, the observation that two metabolites were highly correlated with 99% photocytotoxicity observed in the cell assay (Table 2) suggests that the leaf extracts of parthenium weed possesses potent photosensitisers. Photocytotoxicity requires the presence of light-absorbing molecular features such as a porphyrin ring (found in chlorophyll or its breakdown products) or other conjugated double-bond moieties. The putative molecular formulae and retention times of the entities associated with photocytotoxicity in this study suggest the involvement of breakdown products of chlorophyll; chlorophyll breakdown products containing a porphyrin ring would contain at least four nitrogen atoms and elute at 17–22 min under the chromatographic conditions used in this study.

### 3.3. Biotype Differences

There were no major differences in the toxicity of the extracts of the Clermont and Toogoolawah biotypes of parthenium weed. Previous studies evaluating the allelopathic effects of leaf litter from both these biotypes also reported no differences in the potency of these two biotypes [6]. This suggests that the invasive potential of the two biotypes in Australia is not dependent on phytotoxicity. However, significant differences have been reported in the phytotoxicity of parthenium biotypes from its native and introduced range, with the latter being more active against several plant species [6]. Shi [50] also reported that parthenin and coronopilin concentrations were similar in the Clermont and Toogoolawah biotypes (in the introduced range) while both these compounds were absent in a biotype from Argentina (native range) that possessed another sesquiterpene lactone, hymenin, instead. Picman, et al. [51] reported that most parthenium weed biotypes from its introduced range (Australia, India and South Africa) produced similar sesquiterpene lactones (mainly parthenin and coronopilin) to those present in North American biotypes; however, South American biotypes produced hymenin as their major sesquiterpene lactone. Hence, the authors suggested that most biotypes in the introduced range came from North America [51]. In contrast, South American biotypes of parthenium weed have shown remarkable diversity in their sesquiterpene lactone chemistry [17].

The results of previous studies suggest that the invasion success of parthenium weed is due to the lack of natural enemies in the introduced range, resilient biology and also rapid evolutionary adaptations related to growth and development [1,3]. Recent studies have revealed that prolific seed production, germination ability under harsh conditions, vigorous growth habit, and the capacity to tolerate abiotic stresses through morphological and physiological adaptations are the major factors responsible for the invasion success of parthenium weed, in general, and of the Clermont biotype, in particular [23,25]. Although secondary chemistry plays a significant role in deterring the herbivory of parthenium weed, it appears to be relatively less important in the success of highly invasive biotypes.

## 4. Materials and Methods

### 4.1. Plant Material

Numerous (~20) plants each of the Clermont and Toogoolawah biotypes of parthenium weed were grown in a temperature-controlled glasshouse (30/20 ± 2 °C, day/night with a 12/12-hour photoperiod) during the summer of 2017. The seeds of each biotype were sown in plastic pots (30 × 30 cm, diameter by height) filled with a heavy clay loam soil which was found to be ideal for parthenium weed growth. After the initial seeding, the pots were thinned to one plant per pot. The plants were irrigated as required and were not fertilised. The plants initiated flowering between 50 and 60 days after sowing, and both biotypes reached maximal flowering after 70 days. Six plants of each biotype exhibiting healthy and uniform growth were harvested at this stage. The plants were carefully uprooted from the soil and the leaves, and the shoots and roots were separated with scissors. As parthenium weed has a tap root system, the roots were easily extracted from the soil and were then gently rinsed with distilled water. All the plant material (different parts separately) was immediately placed into re-sealable plastic bags and stored at −20 °C until further processing.

### 4.2. Extraction

Approximately 5 g of each fresh plant tissue (roots, shoots, and leaves) from six individual plants of each biotype were extracted separately, with each plant serving as a replicate. A Buchi Speed Extractor™ (Model E-916, Flawil, Switzerland) was employed, using a programmed extraction method with 100% methanol at 35 °C for 20 min under high pressure, following the manufacturer’s recommended methods for fresh plant materials. The eluent was then collected and dried using rotary evaporation, and following the transfer of the extracts to scintillation vials, was further dried under nitrogen gas before being reconstituted in methanol at a concentration of 10 mg mL^−1^. The crude extracts obtained from different plants of each biotype, and from all plant parts, were individually used for the bioassay and analysed for their metabolites. The experiment analysed two biotypes and three plant tissues, for each of the six plants, for a total of 36 individual samples evaluated in further metabolic profiling studies. Two extracts of each biotype and tissue type were pooled to conduct the phytotoxicity and cytotoxicity bioassays, which were replicated three times and repeated over time.

### 4.3. Evaluation of Phytotoxicity

Methanolic extracts (10 mg mL^−1^) of leaf, shoot and root tissues were serially diluted further in methanol to produce a concentration gradient i.e., 0.12, 0.25, 0.50, 1.00 and 2.00 mg mL^−1^. The extracts were then applied uniformly (0.5 mL) to Whatman No. 1 filter papers in glass Petri dishes (30 mm diameter) and the treatments were replicated three times. A treatment of 0.5 mL methanol alone was the vehicle-only control. After solvent evaporation in a fume cabinet, each dish was individually moistened with 0.5 mL distilled water. Ten seeds of cress (*Lepidium sativum* L.) or annual ryegrass (*Lolium rigidum* Gaud.) were utilised as the seed indicators for the bioassay of phytotoxicity, and were placed on the moistened filter paper in each dish. The dishes were then covered and placed in a germination box that was maintained in a humid environment, after randomization in a completely randomized design. The dishes were incubated at 25 °C in the dark for 24 (garden cress) or 36 (annual ryegrass) hours. After this time, the germination percentage, radicle length and hypocotyl length of germinated seedlings were recorded. The seedlings with a radicle length of >1 mm were considered as having germinated. The percentage inhibition of germination and the length of radicles and hypocotyls relative to the control was calculated [52]. The pure parthenin compound (University of Hohenheim, Germany) was also evaluated against the garden cress at a range of concentrations (200 to 1000 μM).

### 4.4. Evaluation of Cytotoxicity and Photocytotoxicity

The cytotoxicity of methanolic extracts was evaluated against the mouse embryonic fibroblast cell line NIH3T3. Briefly, the cells were expanded in Dulbecco’s modified Eagle medium (DMEM, Life Sciences) containing 10% fetal calf serum (Bovogen, Keilor East VIC, Australia) in 5% carbon dioxide (CO_2_) at 37 °C. The cells were seeded at a rate of 5 × 10^3^ per well in 96-well plates and incubated until 80% confluency was reached. The following day, the crude extracts (10 mg mL^−1^) of the leaf, shoot and root tissues described above were mixed with the cell culture media to produce serial doubling dilutions ranging from 0.0625 to 0.5 mg mL^−1^. As additional experimental controls, the cells maintained in a culture medium or with equivalent volumes of methanol-containing media as a vehicle-only control were also included in the study. A known photosensitising agent, 8-methoxypsoralen (Sigma Aldrich, Castle Hill NSW Australia), was compared at a concentration of 10 µM as a positive appropriate control for comparative photocytotoxicity. The cultures were maintained at 5% CO_2_ and 37 °C for 4 h in the presence of extracts. Each experiment was performed in parallel on two 96 well-plates, with one exposed to UV-A (Vilber Bio-Sun illumination system, Marine, France with the UV dose measured using a Vilber radiometer, while the second plate was maintained in the dark for the same period (sham exposure). A uniform UV-A dose of 2 J cm^−2^ was applied in all experiments.

After the UV-A or sham exposure, the culture medium in all the wells was removed and replaced with a fresh cell culture medium before the plates were returned to the incubator for a further 48 h incubation, prior to the analysis of cytotoxicity using a standard colorimetric 3-(4,5-dimethylthiazol-2-yl)-2,5-diphenyltetrazolium bromide (MTT) assay (Invitrogen, ThermoFisher, Melbourne, Australia). In this bioassay, the yellow MTT reagent was converted to a purple formazan precipitate in metabolically active cells by the mitochondrial dehydrogenases of living cells. The precipitate was then dissolved in dimethyl sulfoxide (DMSO) and the absorbance was measured using a spectrophotometer at 540 nm (Versamax™, Molecular Devices, Sunnyvale, CA, USA). The viability of the treated cells was presented relative to the untreated control. Following the initial screening for cytotoxicity and photocytotoxicity caused by various plant extracts, the experiment was repeated with a wider range of concentrations with only leaf tissue extracts (0.008 to 0.25 mg mL^−1^) to enable the calculation of respective IC_50_ values. The extracts of other tissues (shoot and root) were not evaluated further as they exhibited limited activity in the initial screening experiment.

### 4.5. Metabolic Profiling

A non-targeted metabolic profiling approach was performed to analyse the metabolites contained in parthenium weed extracts of various plant tissues and biotypes. Methanolic extracts (as described above) were filtered through a 0.22 µm polytetrafluoroethylene (PTFE) filter and analysed with an Agilent 1290 Infinity UHPLC system equipped with a quaternary pump, diode array detector, degasser, temperature-controlled column and cooled autosampler compartments and coupled to an Agilent 6530 Q-TOF mass spectrometer with Agilent Dual Jet Stream ionisation source (Agilent Technologies, Melbourne, Australia). The extracts were separated using a C_18_ Poroshell column (2.1 mm × 100 mm, 2.7 µm particle size) (Agilent Technologies, Santa Clara, CA, USA) at 25 °C, which was preceded by a SB-C_8_ guard column (2.1 mm × 12.5 mm, 5 µm particle size) (Agilent Technologies, Santa Clara, CA, USA). The flow rate of the mobile phase was 0.5 mL min^−1^. The column was equilibrated for 40 min prior to analysis. The separation was obtained using a gradient of solvent A (water, 0.1% formic acid) and solvent B (95% acetonitrile, 0.1% formic acid). A stepwise linear gradient was employed commencing with 5% B for 1 min and ramping to 100% B over 16 min, where it remained until 25 min. The Q-TOF was calibrated in positive ion mode with nebuliser gas set at 35 psig, capillary voltage at 3500 V and fragmentor voltage at 135 V. Nitrogen was used as the drying gas at 250 °C at a flow of 9 L min^−1^.

The metabolites were identified by comparison with known standards and by matching the molecular features and molecular formulae as previously reported in the literature (Table 3). The absolute quantitation of parthenin in all the extracts was determined by comparison with a standard curve prepared from technical grade parthenin. However, the relative abundance of other metabolites present was determined by annotation with the comparison of known molecular features as reported in the literature. Molecular features were extracted from data files using Profinder (ver. 8.0, Agilent Technologies, Melbourne, Australia), and the graphical presentation of metabolomics data was generated by Mass Profiler Professional (ver. 13.0, Agilent Technologies, Melbourne, Australia).

### 4.6. Statistical Analyses

The data were subjected to a factorial analysis of variance to evaluate the significance of the treatment factors and interaction effects using Statistix (ver. 8.1, Tallahassee, FL, USA). The percent inhibition of seed germination, radicle length, hypocotyl length, and cell death was regressed against the log of the extract concentrations using a linear regression model in SigmaPlot (ver. 14, Chicago, IL, country). The extract concentrations responsible for the 50% inhibition of the total response (IC_50_) were estimated for various parameters using generated regression equations. The statistical relationships between the metabolites separated via LC–MS and the respective bioactivity were tested using stepwise linear regression via SPSS (ver. 24, IBM Corporation, Chicago, IL USA). All the models used forward selection with no components starting in the model, with *p* < 0.05 for the variables entering the model and *p* > 0.1 for variables leaving the model.

## Figures and Tables

**Table 1:** Correlation between the abundance of the known phytotoxic compounds from parthenium weed and the three measures of phytotoxicity in the laboratory. Values with asterisks indicate the components which were found to be statistically significantly correlated with the indicated toxicity measures via stepwise linear regression.

			Correlation with Phytotoxicity Measures		
Compound	Formula	Accurate Mass	Germination	Radicle Length	Hypocotyl Length
Parthenin	C_15_H_18_O_4_	262.1140	−0.57	−0.41	−0.43
Ambrosin	C_15_H_18_O_3_	246.1256	−0.72 *	−0.54	−0.46
Coronopilin	C_15_H_20_O_4_	264.1362	−0.67	−0.47	−0.49
Damsin	C_15_H_20_O_3_	248.1412	−0.62	−0.55	−0.37
Chlorogenic acid	C_16_H_18_O_9_	354.0951	−0.60	−0.63 *	−0.61

**Table 2:** Results of the stepwise linear regression analysis between the activity bioassays and the composition of the extracts as determined by the LC–MS QTOF analysis. Accurate masses, retention times and the molecular formulae correspond to the compound in boldface type in the first column.

Significant Compounds	Coefficient of Determination (R^2^)	Accurate Mass	Retention Time (min)	Molecular Formula
**Germination inhibition**				
Compound **187**	0.75	430.2276	8.8	C_15_H_34_N_4_O_10_
Compound 187 + **124**	0.92	341.1825	8.1	C_15_H_25_N_4_O_5_
**Radicle elongation inhibition**				
Compound **541**	0.74	290.1880	12.4	C_18_H_26_O_3_
Compound 541 + **46**	0.93	297.1576	5.2	C_15_H_23_NO_5_
**Hypocotyl elongation inhibition**				
Compound **612**	0.61	352.2614	13.9	C_21_H_36_O_4_
Compound 612 + **1048**	0.84	914.6030	21.1	C_56_H_75_N_6_O_5_
Compound 612 + 1048 + **840**	0.92	582.4118	19.0	C_30_H_50_N_10_O_2_
**Cytotoxicity**				
Compound **19**	0.74	218.0188	0.86	C_7_H_2_N_6_O_3_
Compound 19 + **32**	0.89	281.1825	1.1	C_10_H_25_N_4_O_5_
**Photocytotoxicity**				
Compound **1242**	0.84	580.3956	20.3	C_30_H_48_N_10_O_2_
Compound 1242 + **1075**	0.99	402.2272	17.9	C_21_H_30_N_4_O_4_

**Table 3:** List of the major secondary metabolites reported from parthenium weed classified by chemical group and biological activities.

Compound	Effect	Reference
**Sesquiterpene lactones**		
Parthenin	Phytotoxic	[8,10,11,13]
	Cytotoxic, allergenic	[16,23,45,47]
Ambrosin	Allergenic/dermatitis	[16,17,18,19]
Coronopilin	Phytotoxic	
Damsin	Allelopathic	
Hysterin	Allelopathic	
Hymenin	-	
**Phenolics**		
Caffeic acid	Phytotoxic	[8,19]
p-coumaric acid		
Anisic acid		
Vanillic acid		
Ferulic acid		
Fumaric acid		
Aerulic acid		
p-hydroxybenzoic acid		
Chlorogenic acid		
Scopoletin	Cytotoxic	[49]
**Flavonoids**		
Kaempferol	Not known	[20]
Quercetin 3-o-glycosides		
Quecetagetin 3,7-dimethyl ether		

## Supplementary Materials

The following are available online at https://www.mdpi.com/2072-6651/12/7/447/s1, Table S1: Analysis of variance for the effect of the shoot and root extracts of the parthenium weed biotypes on germination, radicle elongation and hypocotyl elongation inhibition of garden cress seeds, Table S2: Analysis of the variance for the effect of the leaf extracts of the parthenium weed biotypes on the germination, radicle elongation and hypocotyl elongation inhibition of the garden cress and annual ryegrass seeds, Table S3: Analysis of variance for the effect of the leaf, shoot and the root extracts of the parthenium weed biotypes on the inhibition of the NIH3T3 murine fibroblasts in the absence (cytotoxicity) or presence of UV-A radiation (photocytotoxicity), Table S4: Analysis of the variance for the effect of the extended concentrations of leaf extracts (second experiment) of parthenium weed biotypes on the inhibition of NIH3T3 murine fibroblasts in the absence (cytotoxicity) or presence of UV-A radiation (photocytotoxicity), Table S5: Analysis of the variance for the parthenin quantities and the relative abundance of the other major compounds in the leaf, shoot or root extracts of two parthenium weed biotypes detected in this study.
