# Supplementary material for: Toxic Potential and Metabolic Profiling of Two Australian Biotypes of the Invasive Plant Parthenium Weed (Parthenium hysterophorus L.)
Source: Toxins (Basel). 2020 Jul 10;12(7):447. doi: 10.3390/toxins12070447 (PMC7404986; doi:10.3390/toxins12070447)
Supplement: Supplementary file 1 [file toxins-12-00447-s001.zip › toxins-851288-supplementary.pdf]

Supplementary Materials: Toxic Potential and Metabolic Profiling of Two Australian Biotypes of the Invasive Plant Parthenium Weed (*Parthenium hysterophorus* L.)

Ali Ahsan Bajwa, Paul A. Weston, Saliya Gurusinghe, Sajid Latif, Steve W. Adkins and Leslie A. Weston

Table S1. Analysis of the variance for the effect of shoot and root extracts of parthenium weed biotypes on the germination, radicle elongation and the hypocotyl elongation inhibition of garden cress seeds.

Source of Variation	Degree of Freedom	*P*-Values					
		Shoot Extracts			Root Extracts		
		GI	RI	HI	GI	RI	HI
Biotype (B)	1	0.823	0.548	0.239	0.354	0.793	0.959
Concentration (C)	4	0.964	0.899	0.738	0.473	0.311	0.559
B × C	4	0.692	0.936	0.974	0.436	0.727	0.380

GI = germination inhibition, RI = radicle inhibition, HI = hypocotyl inhibition. The p values < 0.05 are significant.

Table S2. Analysis of the variance for the effect of the leaf extracts of parthenium weed biotypes on the germination, radicle elongation and the hypocotyl elongation inhibition of garden cress and annual ryegrass seeds.

Source of Variation	Degree of Freedom	*P*-Values					
		Garden Cress			Annual Ryegrass		
		GI	RI	HI	GI	RI	HI
Biotype (B)	1	0.002	0.089	0.010	0.151	0.343	0.428
Concentration (C)	4	<0.001	<0.001	<0.001	<0.001	<0.001	<0.001
B × C	4	0.466	0.556	0.185	0.953	0.873	0.954

GI = germination inhibition, RI = radicle inhibition, HI = hypocotyl inhibition. The p values < 0.05 are significant.

Table S3. Analysis of the variance for the effect of the leaf, shoot and root extracts of the parthenium weed biotypes on the inhibition of NIH3T3 murine fibroblasts in the absence (cytotoxicity) or presence of UV-A radiation (photocytotoxicity). The p values < 0.05 are significant.

Source of Variation	Degree of Freedom	*P*-Values	
		No UV-A Exposure	UV-A Exposure
Tissue (T)	2	<0.001	<0.001
Biotype (B)	1	<0.001	0.644
Concentration (C)	3	<0.001	<0.001
T × B	2	0.125	0.259
T × C	6	0.002	0.115
B × C	3	0.942	0.855
T × B × C	6	0.711	0.948

Table S4. Analysis of the variance for the effect of the extended concentrations of leaf extracts (second experiment) of the parthenium weed biotypes on the inhibition of NIH3T3 murine fibroblasts in the absence (cytotoxicity) or presence of UV-A radiation (photocytotoxicity). The p values < 0.05 are significant.

Source of Variation	Degree of Freedom	*P*-Values	
		No UV-A Exposure	UV-A Exposure
Biotype (B)	1	0.858	0.107
Concentration (C)	5	<0.001	<0.001
B × C	5	0.741	0.795

Table S5. Analysis of the variance for the parthenin quantities and the relative abundance of other major compounds in the leaf, shoot or the root extracts of the two parthenium weed biotypes detected in this study. The p values < 0.05 are significant. Relative abundance data were subjected to square-root transformation ($\sqrt{(x + 0.5)}$ before analysis. The p values < 0.05 are significant.

Source of Variation	Degree of Freedom	*P*-Values				
		Quantity	Relative Abundance			
		Parthenin	Coronopilin	Ambrosin	Damsin	Chlorogenic acid
Tissue (T)	2	<0.001	<0.001	<0.001	<0.001	0.575
Biotype (B)	1	0.382	0.197	0.133	0.231	0.297
T × B	2	0.282	0.485	0.046	0.609	0.445
